# Supplementary material for: Inorganic Polyphosphate Modulates Chromosome Transmission Fidelity in the Fission Yeast Schizosaccharomyces pombe
Source: Biomolecules. 2025 Sep 18;15(9):1331. doi: 10.3390/biom15091331 (PMC12467933; doi:10.3390/biom15091331)
Supplement: Supplementary file 1 [file biomolecules-15-01331-s001.zip › Supplementary Resubmission.pdf]

Supplementary

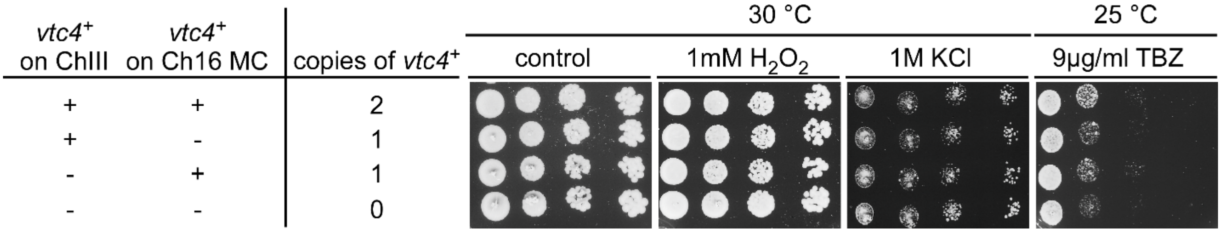

Supplementary Figure S1. *vtc4⁺* gene dosage does not alter the growth of Ch16 MC strains.

Serial dilution patch assay (10^4 to 10^1 cells) of *S. pombe* MC Ch16 strains carrying different copy numbers of *vtc4⁺*: strain with two copies (*vtc4⁺* on both endogenous chromosome III and the Ch16 MC; ++), strains with one copy of *vtc4⁺* either on chromosome III or the Ch16 MC(+-/-+) and a Ch16 MC strain where both *vtc4⁺* ORFs had been deleted (--). Cells were spotted on YE5S medium and incubated for 4 days at the indicated temperatures. Plates were supplemented with 1 mM H_2O_2 (oxidative stress), 1 M KCl (osmotic stress), or 9 µg/mL thiabendazole (TBZ, microtubule poison).

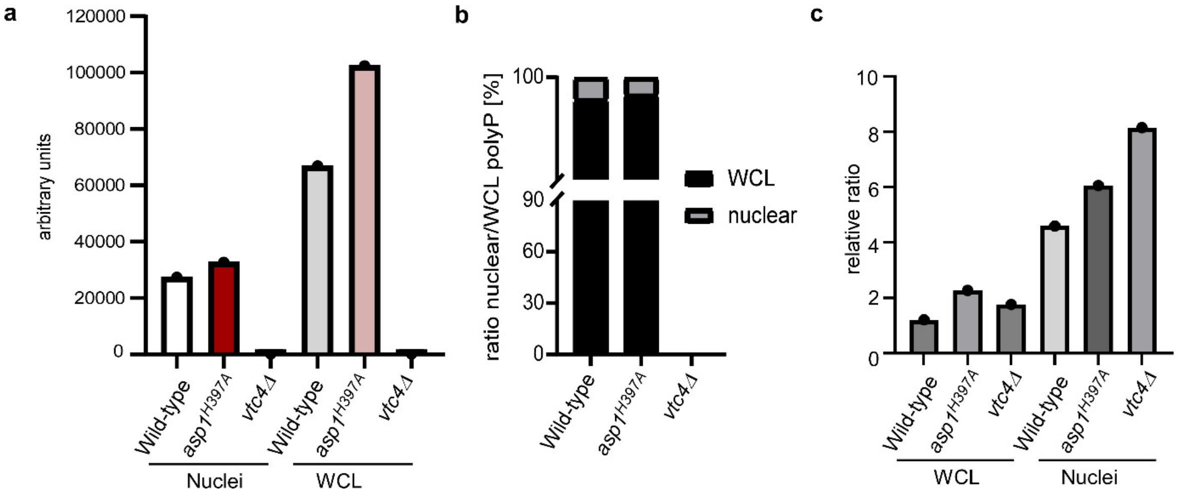

Supplementary Figure S2. Quantification of polyP in nuclear extracts**.**

(a) Densitometric analysis of polyP signals from native PAGE gels of whole cell lysates (WCL) and nuclear extracts (nuclei). Strains analyzed were wild-type strain, the *asp1^{H397A}* strain, which has higher than wild-type 1-IPP levels and thus increased polyP and a strain with a deletion of *vtc4⁺* (*vtc4Δ*). Band intensities were quantified using the Analyze Gel function in ImageJ. **(b)** Ratio of polyP signal intensity in nuclear extracts relative to WCL for the indicated strains. **(c)** Quantification of western blot analysis of WCL and isolated nuclei from wild-type, *asp1^{H397A}* and *vtc4Δ S. pombe* cells. Anti H3 antibody (mainly nuclear) and anti-GAPDH-antibody (mainly cytoplasmic) were used. The H3 to GAPDH signal ratios were quantified using ImageJ. WCL were lysed using glass beads and nuclei were enriched by differential centrifugation using Ficoll. Protein extracts were separated on 4–12% Bis-Tris gels and transferred to PVDF membranes. Membranes were probed with antibodies against GAPDH recognizing Tdh1 and histone H3

recognizing H3.1, H3.2. and H3.3.

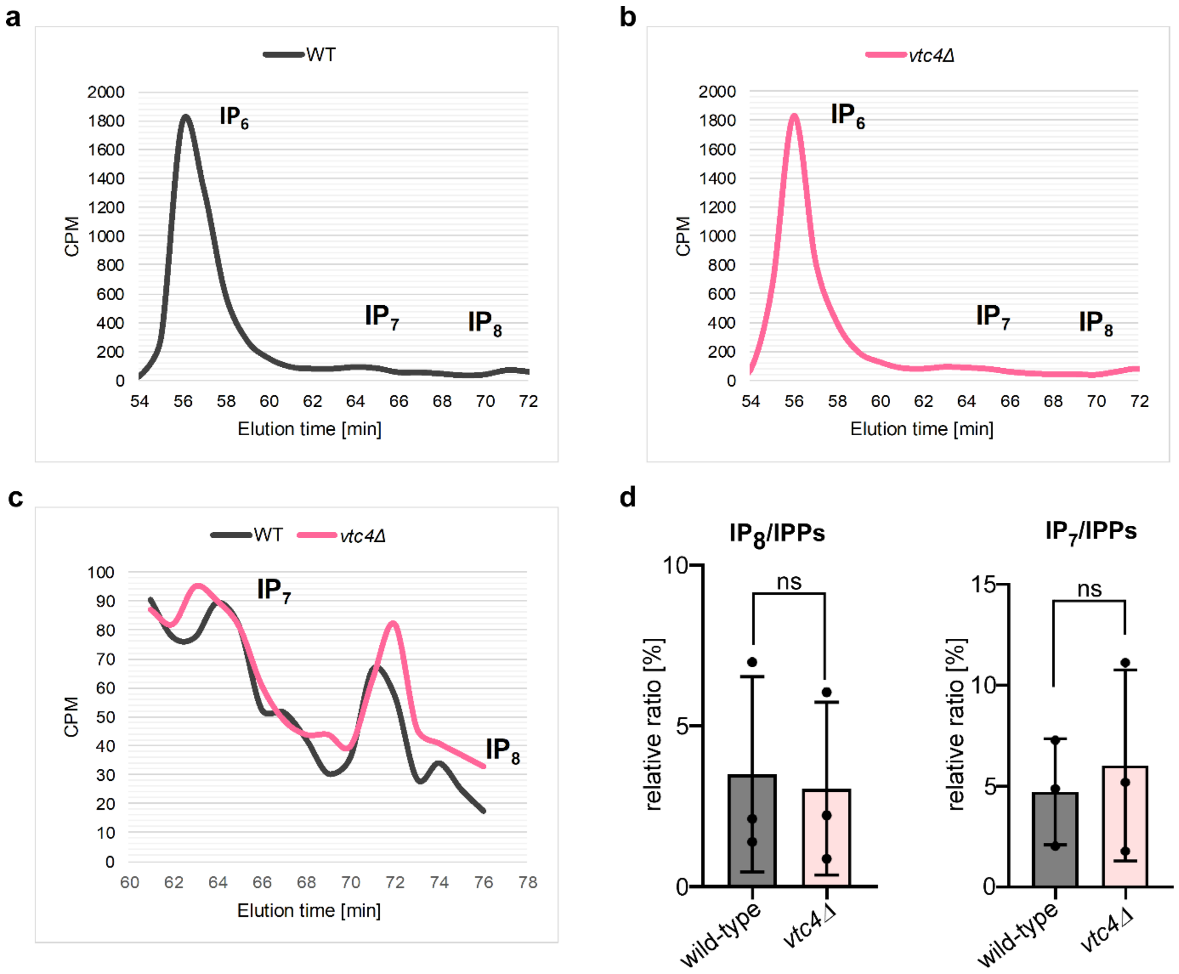

Supplementary Figure S3. PolyP has no influence on inositol pyrophosphate levels. **(a-c)** SAX-HPLC elution profiles of wild-type (WT) and *vtc4Δ* cells.

(a) Representative chromatogram of WT cells. **(b)** Representative chromatogram of *vtc4Δ* cells. **(c)** Close-up of the IP_7 and 1,5-IP_8 peaks from (a) and (b). **(d)** Quantification of inositol pyrophosphate species. Relative levels of IP_7 and 1,5-IP_8 were calculated as a percentage of total inositol phosphates (IP_6, IP_7, and 1,5-IP_8). Data represent mean values ± SD from three independent measurements. Statistical analysis was performed using an unpaired two-tailed Student's t-test with Welch's correction. CPM, counts per minute.

temperature	strain		
	vtc4Δ	*fta2-291*	*vtc4Δ fta2-291*
25 °C	++	++	++
31 °C	++	+	++/+
32 °C	++	-	+
	vtc4Δ	*mis15-68*	*vtc4Δ mis15-68*
25 °C	++	++	++
30 °C	++	++	++
33 °C	++	+/-	+
> 33 °C	n.d.	n.d.	n.d.
	vtc4Δ	*mis6-302*	*vtc4Δ mis6-302*
25 °C	++	++	++
30 °C	++	+	+
31 °C	++	+/-	+/-
≥ 32 °C	++	-	-

Supplementary Figure S4. Loss of Vtc4 mediated polyP synthesis partially rescues the temperature sensitivity of the *mis15-68* but not the *mis6-302* strain*.*

Summary table of serial dilution patch assay (10^4 to 10^1 cells) of *S. pombe mis15-68* and *mis6-302* single and *mis15-68 vtc4Δ* and *mis6-302 vtc4Δ* double mutant strains*.* Cells were spotted on YE5S medium and incubated for 4 days at the indicated temperatures. Growth categories are indicated as follows: ++, good growth; +, slightly reduced growth; +/−, reduced growth; −, no growth.

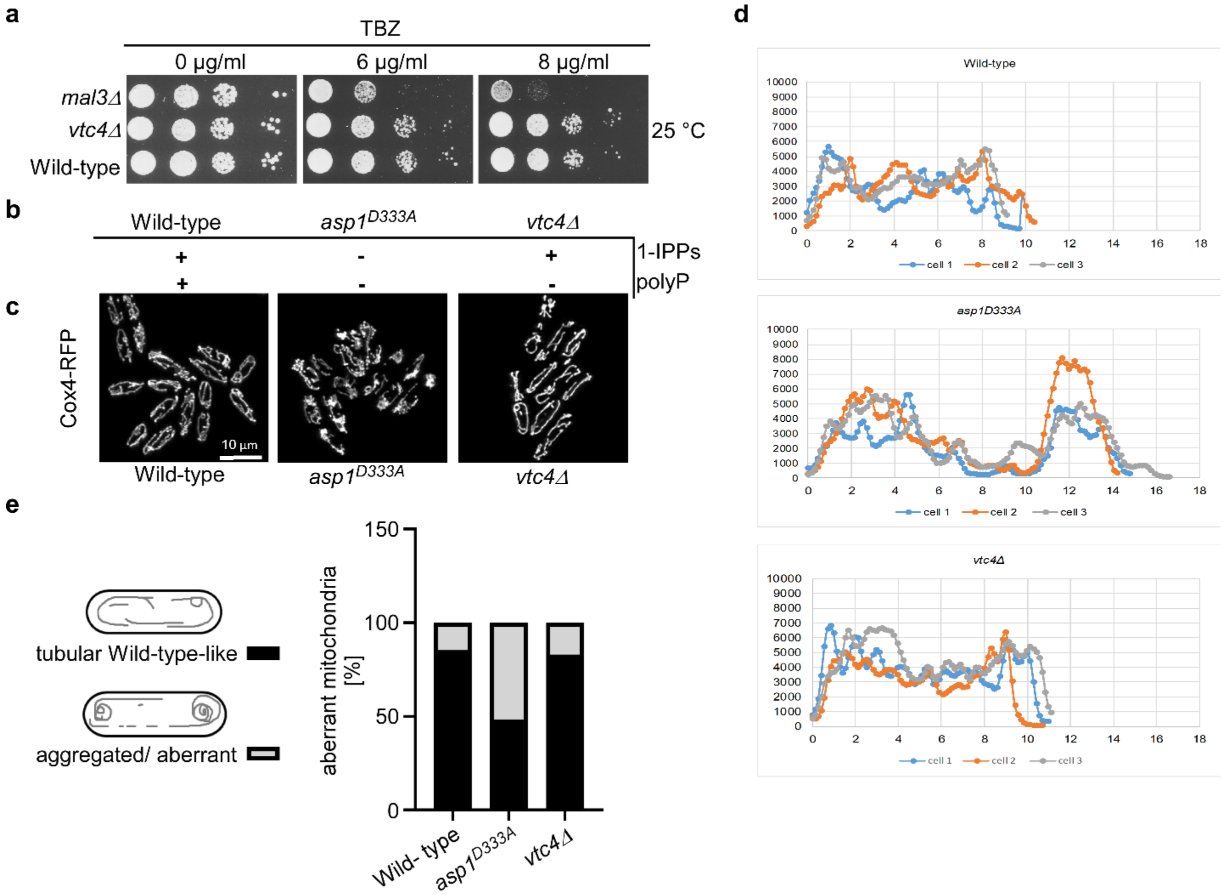

Supplementary Figure S5. PolyP absence does not affect TBZ-sensitivity or the mitochondria-microtubule crosstalk.

(a) Serial dilution patch test of the indicated *S. pombe* strains grown on minimal medium (MM) with appropriate supplements and with 0, 6, or 8 µg/mL thiabendazole (TBZ). Serial dilutions (10^4 to 10^1 cells) patch tests of the indicated strains were incubated at 25 °C for 6 days. The *mal3Δ* strain (lacking the microtubule plus-end binding protein EB1) (Beinhauer et al. 1997) served as a TBZ-sensitive positive control. **(b)** Diagrammatic representation of presence of 1-IPPs and polyP in the wild-type, *asp1*^{D333A}, and *vtc4Δ* strains. +: present; -: absent. **(c)** Live-cell fluorescence microscopy of Cox4-RFP (inner mitochondrial membrane) signals of the indicated strains incubated at 30 °C in liquid medium. Scale bar: 10 µm. **(d)** Cox4-RFP intensity profiles of three representative cells per indicated genotype. Signal distribution was measured along the longitudinal axis of each cell using the *Plot Profile* tool in ImageJ, which calculates average grey values per pixel column within a rectangular selection. **(e)** Quantification of mitochondrial morphology from (c). A minimum of 29 interphase cells/strain with a length of 10-14 µm were analyzed. Mitochondria were classified as wild-type-like (black bars) or aberrant/aggregated (gray bars) based on shape and network continuity. Bars indicate the percentage of cells with the respective phenotype.

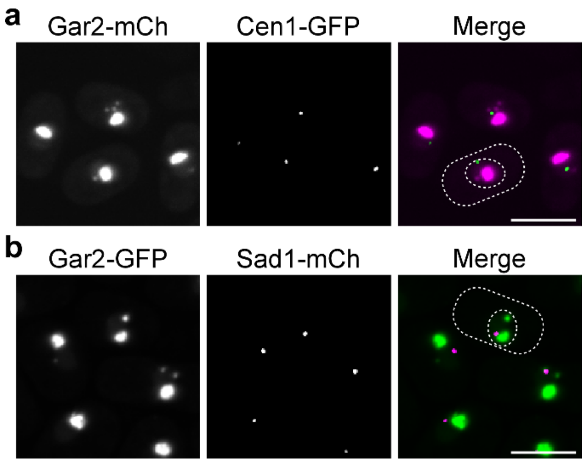

Supplementary Figure S6. Localization of Gar2 in cells grown on solid media.

(a) Live-cell photomicrographs of cells expressing endogenous Gar2-mCherry and LacO-GFP (close to centromere I) **(b)** Live-cell photomicrographs of cells expressing endogenous Gar2-GFP and the spindle pole body component Sad1-mCherry. Dashed lines display the cell (cylindrical) and nuclear (round) boundaries. Scale bars: 5 µm.

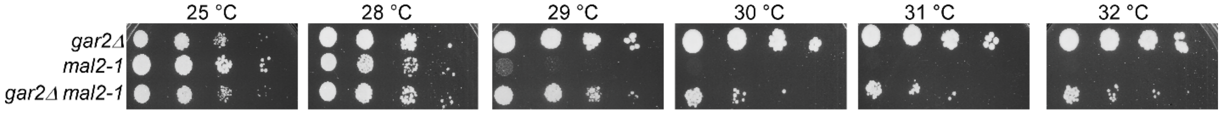

Supplementary Figure S7. Deletion of *gar2*$^{+}$ (*gar2Δ*) partially suppresses the temperature sensitivity of the *mal2-1* strain.

Serial dilution patch test of indicated strains (10^4-10^1 cells), spotted onto YE5S plates and incubated for 4 days at shown temperatures.
