# Supplementary material for: Inorganic Polyphosphate Modulates Chromosome Transmission Fidelity in the Fission Yeast Schizosaccharomyces pombe
Source: Biomolecules. 2025 Sep 18;15(9):1331. doi: 10.3390/biom15091331 (PMC12467933; doi:10.3390/biom15091331)
Supplement: Supplementary file 1 [file biomolecules-15-01331-s001.zip › Table S1-S3 Resubmission.pdf]

Table S1. Strains used in this work.

Reference	Genotype	Source
UFY103	h^- ade6-M210 leu1-32 ura4-D6 Ch16 MC [ade6-M216]	M. Yanagida
UFY221	h^+ LacI-GFP:$his7^+$ LacO-repeat:$lys1^+$ leu1-32 ura4-Dx	M. Yanagida
UFY518	h^- fta2-GFP:kanR ade6-M210 leu1-32 ura4-D6	Lab collection
UFY605	h^- his3-D1 ade6-M210 leu1-32 ura4-D18	K. Gould
UFY606	h^+ his3-D1 ade6-M210 leu1-32 ura4-D18	K. Gould
UFY852	h^- mal2-1 leu1-32 ade6-M210 ura4-D18	Lab collection
UFY1024	h^- spc7-23:$his3^+$ his3-D1 ade6-M210 leu1-32 ura4-D18	Lab collection
UFY1048	h^- fta2-291:$his3^+$ $his3^-$ leu1-32 ura4-D18 ade6-M210	Lab collection
UFY1049	h^+ fta2-291:$his3^+$ $his3^-$ leu1-32 ura4-D18 ade6-M210	Lab collection
UFY1065	h^- mis15-68 leu1-32 ura4-D18 ade6-M210	M. Yanagida
UFY1156	h^- asp1Δ::kanR his3-D1 ade6-M216 leu1-32 ura4-D18	Lab collection
UFY1158	h^- mal3Δ::$his3^+$ $his3^-$ ade6-M210 leu1-32 ura4-D18	Lab collection
UFY1503	h^+ mis6-302 leu1-32 ura4-D18 ade6-M210	I. Hagan
UFY1579	h^+ $asp1^{H397A}$:kanR his3-D1 ade6-M210 leu1-32 ura4-D18	Lab collection
UFY2118	h^- sad1-mCherry:kanR leu1-32	M. Yanagida
UFY2908	h^- cox4-RFP:LEU2 ade6-m210 leu1-32 ura4-D18	P. Tran
UFY3033	h^- vtc4Δ::kanR his3-D1 ade6-M210 leu1-32 ura4-D18	Lab collection
UFY3063	h^+ vtc4Δ::kanR his3-D1 ade6-M210 leu1-32 ura4-D18	Lab collection
UFY3227	h^- leu1-32 ura4-D18 $gar2^+$-mCherry:kanR	M. Yanagida
UFY3354	h^- gar2Δ::KanMX6 leu1-32 ura4-D18	X. He
UFY3394	h^+ fta2-291:$his3^+$ vtc4Δ::kanR leu1-32 ade6-M210 ura4-D18	This work
UFY3535	h^+ gar2Δ::KanMX6 leu1-32 ura4-D18	This work
UFY3539	h^+ gar2Δ::KanMX6 fta2-291:$his3^+$ leu1-32 ura4-D18 his3-D1 ade6-M210	This work
UFY3540	h^- gar2Δ::KanMX6 mal2-1 leu1-32 ura4-D-18 ade6-M210	This work
UFY3581	$h^?$ vtc4Δ::KanMX6 ade6-M210 leu1-32 ura4-D-18 Ch16 MC [ade6-M216]	This work
UFY3593	h^- gar2Δ::KanMX6 ura4-D18 leu1-32 ade6-M210 his3-D1	This work
UFY3693	$h^?$ vtc4Δ::hphMX6 ade6-M210 leu1-32 ura4-D-18 Ch16 MC [ade6-M216]	This work
UFY3694	$h^?$ vtc4Δ::KanMX6 ade6-M210 leu1-32 ura4-D-18 Ch16 MC [vtc4Δ::hphMX6 ade6-M216]	This work
UFY3352	h^+ gar2-GFP:KanMX6 ade6-210 leu1-32 ura4D-18	X. He
UFY3360	h^+ LacI-GFP:$his7^+$ LacO-repeat:$lys1^+$ gar2-mCh::kanR leu1-32 ura4-D-18	This work
UFY3364	h^- sad1-mCherry:kanR gar2-GFP:kanR leu1-32	This work
UFY3604	h^- cox4-RFP:LEU2 vtc4Δ::kanMX ade6-M210 leu1-32 ura4-D18	This work
UFY3606	h^- cox4-RFP:LEU2 $asp1^{D333A}$:kanMX ade6-M210 leu1-32 ura4-D18	This work
UFY3787	h^- spc7-23:$his3^+$ vtc4Δ::kanMX his3-D1 ade6-M210 leu1-32 ura4-D18	This work
UFY3791	h^- mis15-68 vtc4Δ::kanMX leu1-32 ura4-D18 ade6-M210	This work
UFY3795	h^- mis6-302 vtc4Δ::kanMX leu1-32 ura4-D18 ade6-M210	This work

Please note that alleles on the Ch16 MC are shown in brackets []

Table S2. Oligonucleotides used in this work

**Description**	**Sequence**
5‘ genome integration *vtc4Δ::hygR*	TTATGCATTGAAGTTTAAGATAACATTGGCGCTGATTGCAGCAAAAGTAATCACGCATATAGCTGTAGGACCAAAACGATCGACATGGAGGCCCAGAATAC
3’ genome integration *vtc4Δ::hygR*	ATGAAGTTTGGTCAGCTTTTGAAGGAAACGTTGATGTATGAATACAAGTATTCATATGTCAACTATGACAAACTAAAGAATGGATGGCGGCGTTAGTATCG
5’ UTR verification *vtc4Δ::hygR* fwd	CTCCACCACACCTGCAAAC
3’ UTR verification *vtc4Δ::hygR* rev	CCCCGTTGACCATGTTCCC

Table S3. Plasmids used in this work

**Description**	**Source**
pJR2-3XL	[48]
pFA6a-*hphMX6*	[45]
pJR2-3XL-*vtc4*$^{+}$	This work
